# Miniature Robotic Swimmer with Precise 2D Motion Control via Acoustic Vortex‐Induced Propulsion

**DOI:** 10.1002/advs.202515389

**Published:** 2025-12-12

**Authors:** Chadi Ellouzi, Nicholas Andrianto, Glen Vosgerichian, Farhood Aghdasi, Joshua Lloyd, Ali Zabihi, Chen Shen

**Affiliations:** ^1^ Department of Mechanical Engineering Rowan University Glassboro NJ 08028 USA

**Keywords:** acoustic vortex, acoustofluidics, propulsion, robotic swimmer, ultrasound

## Abstract

The development of advanced robotic systems capable of precise movement without relying on traditional mechanical actuators is a growing area of research. One promising approach involves the use of acoustic waves, where sound waves are used to generate a propulsion force without the use of any moving parts. However, achieving controlled 2D movement in such systems remains a challenge, particularly in terms of efficiency, precision, and scalability. This paper explores the use of 3D‐printed focused acoustic vortex propulsion (FAVP) lenses to drive a miniature robotic swimmer in two dimensions. The principles behind acoustic vortex generation and its application to create both rotational and translational motion on the miniature robot are investigated. The findings demonstrate that a specially designed acoustic lens can focus sound waves to produce localized vortices and streaming forces, which are then harnessed for precise 2D motion control. The robotic swimmer is tested in a variety of controlled environments to validate its ability to perform complex maneuvers, such as forward motion, rotational control, and directional steering. This research highlights the potential of acoustic vortex propulsion as a viable solution for non‐contact, high‐precision movement in small‐scale robots, with profound implications in fields such as micro‐robotics and underwater exploration.

## Graphical abstract

In this work, a miniature robotic swimmer achieves precise 2D motion using sound waves with customized 3D‐printed lenses. Localized acoustic vortices drive both translation and rotation of the swimmer without mechanical parts. This contactless approach offers a novel route to controlled micro‐scale movement, with promising applications in biomedical devices, micro‐robotics, and underwater exploration.

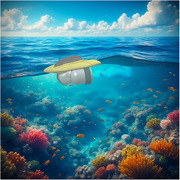

## 1 Introduction

In recent years, the field of robotics has made significant strides, with innovations focusing on reducing the complexity and increasing the precision of robotic systems.^[^ ^1^, ^2^, ^3^ ^]^ Traditional actuators, like motors and propellers, are commonly used in robotic systems for their high propulsion efficiency.^[^ ^4^, ^5^ ^]^ However, they come with significant challenges in the micro‐robotics field due to difficulties in miniaturization, mechanical complexity, and energy efficiency.^[^ ^6^, ^7^ ^]^ The use of mechanical systems with moving parts also makes them susceptible to failure and undesirable in certain scenarios, such as military applications. As such, one of the most exciting areas of research is the development of non‐movable part propulsion mechanisms, particularly in the micro‐robotics field.^[^ ^8^, ^9^, ^10^, ^11^ ^]^ To tackle the aforementioned challenges, researchers have begun to explore alternative propulsion methods, such as magnetic,^[^ ^12^, ^13^ ^]^ optical^[^ ^13^, ^14^ ^]^ chemical^[^ ^15^, ^16^, ^17^ ^]^ approaches. Among these, acoustic propulsion has emerged as a promising method, leveraging the unique properties of sound waves to generate force and movement in small‐scale systems.^[^ ^18^, ^19^ ^]^ By designing the actuation and receiving components, precise force and torque can be generated on objects, providing a mechanism for movement without direct contact.^[^ ^20^, ^21^ ^]^

Recent research has demonstrated the feasibility of using acoustic fields for propulsion in various fluid environments. For instance, Luo et al. introduced micro‐robotic swimmers inspired by biological systems, powered by microstreaming flows from oscillating air bubbles activated by 300 kHz ultrasound. This setup enables both translational and rotational movement.^[^ ^22^ ^]^ Similarly, Feng et al. developed a swimmer by trapping a gas bubble in a microchannel, using 10 kHz ultrasound to generate propulsion at speeds of up to 45 mm s^−1^.^[^ ^23^ ^]^ Other studies also demonstrated acoustically powered microrobots capable of unidirectional surface‐slipping locomotion, using a bullet‐shaped design with a spherical air bubble inside. Acoustic waves at 237 kHz resonated the bubble to generate propulsion.^[^ ^24^ ^]^ Following the same approach, various studies have proposed different methods for maneuvering microrobots using acoustic wave excitation, including sharp edge propulsion, which facilitates microrobot movement by oscillating a tail similar to a sharp edge. For instance, House et al. introduced a double‐jointed, flagella‐like flapper engineered for whip‐like, non‐reciprocal motion. These flappers were activated at frequencies between 15 and 650 kHz to control and propel the microrobot.^[^ ^25^ ^]^ Similarly, a helical wave propulsion system inspired by both prokaryotic and eukaryotic microorganisms was proposed for driving and controlling a microrobot. To power the helical‐shaped tail of the microrobot immersed in silicon oil, a two‐phase stepper motor operating at frequencies from 4 to 7 kHz was placed at the head of the prototype.^[^ ^26^ ^]^ Other studies used direct surface acoustic waves (SAWs) and bulk acoustic waves (BAWs) in order to generate the needed thrust to drive microrobots or swimmers. For instance, Nishio et al. used a PZT transducer that produced bulk waves at 1.6 MHz to propel a polystyrene swimmer through the concept of acoustic radiation forces. The PZT transducer was mounted at the rear of the swimmer to effectively redirect the acoustic forces required for its movement.^[^ ^27^ ^]^ Similarly, other studies have suggested a swimmer that utilizes acoustic propulsion, powered by SAW, to generate thrust in water. To propel the swimmer, a Rayleigh wave was excited at 9.6 MHz using a 128° Y‐rotated X‐propagation lithium niobate U‐IDT actuator.^[^ ^28^ ^]^ Other studies, using the same concept, introduced a submerged SAW IDT transducer based on the SiO2/Al/LiNbO3 structure. Two IDTs were mounted at the back of the swimmer to generate acoustic waves in opposite directions with respect to the axial axis of the swimmer at a resonance frequency of 19.25 MHz, enabling forward motion.^[^ ^29^ ^]^

To position the proposed system within the broader landscape of microswimmer research, it is important to consider the strengths and limitations of alternative actuation strategies. Chemical propulsion offers autonomous motion and relatively high speeds via catalytic reactions, but suffers from limited control, fuel dependency, and compatibility issues in biological environments.^[^ ^30^, ^31^ ^]^ Optical methods provide high spatial precision and can be integrated with imaging systems, yet their effectiveness is confined to transparent media and constrained by line‐of‐sight requirements.^[^ ^14^, ^30^ ^]^ Electric actuation enables fine control in conductive fluids but often requires electrodes and can introduce undesirable electrochemical effects.^[^ ^30^, ^32^ ^]^ Magnetic systems are widely used due to their deep penetration and non‐invasive nature, particularly in biological contexts; however, they typically rely on external magnetic fields and complex swimmer geometries, which can hinder scalability and individual control.^[^ ^30^, ^33^ ^]^ In contrast, acoustic propulsion presents a compelling balance of precision, remote controllability, and biocompatibility. Acoustic fields can penetrate dense or opaque media, function effectively in confined environments, and generate substantial forces without physical contact.^[^ ^30^, ^34^ ^]^ These characteristics make acoustic methods particularly attractive for microscale robotics, especially when designed to enable directional control and on‐board actuation.

While existing studies have begun to explore the potential of acoustic waves for propulsion and control of microrobotics and swimmers, several gaps remain. Most works only demonstrate a simple type of motion (e.g., translation or rotation) with a limited degree of freedom, which prevents the widespread application of this technology for miniature robots in real‐world settings. A robust method to achieve stable, precise 2D motion in complex, unstructured environments remains scarce. In particular, achieving fine control over both translational and rotational movement in two dimensions is a difficult task, as the forces generated by acoustic propulsion systems for microrobotics applications tend to lack the needed power. In the meantime, those designed for swimmers generally do not offer the level of control necessary for executing complex movement patterns. Moreover, many acoustic methods rely on external transducers to generate acoustic waves, rather than integrating them within the system.^[^ ^35^, ^36^ ^]^ Such approaches require delivering precise acoustic fields on the objects, which can be difficult in complex or open environments where the use of external transducers may not always be feasible, and the generation of desired pressure fields becomes challenging. Therefore, integrating the transducers directly onto the object itself would offer greater convenience, improved utility, and more precise control, paving the way for more advanced and versatile microrobotic systems.

In this work, a miniature robotic swimmer capable of executing stable trajectories on a water surface is presented. The propulsion system employs ultrasound wave transducers coupled with a 3D‐printed, focused acoustic vortex lens, while a microcontroller manages the thrust generation, enabling precise control and complex motion. Acoustic vortices, characterized by their helical wavefronts, carry orbital angular momentum (OAM) that imparts a distinctive doughnut‐shaped intensity profile with a low‐pressure region at the center.^[^ ^37^, ^38^ ^]^ The unique OAM property allows acoustic vortices to transfer energy efficiently to materials or objects through wave‐matter interactions, facilitating energy transfer compared to planar waves.^[^ ^39^, ^40^, ^41^ ^]^ This capability makes them effective for thrust generation. In this design, the adoption of a focusing mechanism to the vortex generation enhances precision by concentrating energy at specific focal points,^[^ ^42^ ^]^ which further helps to stabilize the output motion. This focused energy profile provides a robust thrust, enabling the miniature robotic swimmer to perform complex maneuvers with improved control and efficiency. Through the combination of 3D printing, acoustic vortex propulsion, and real‐time control algorithms, this study aims to overcome the current limitations in achieving scalable, efficient 2D motion for mini‐robots, paving the way for more advanced applications in micro‐robotics and bioengineering.

## 2 Numerical Analysis

### 2.1 Acoustofluidic Propulsion Principle

The acoustofluidic propulsion principle presented in this study is achieved through a focused acoustic vortex beam generated by a piezoelectric transducer coupled with a 3D printed acoustic lens. This configuration produces a vortex pressure field in the fluid, enabling motion without any mechanical components in direct contact with the water. The acoustic driving force *F_ADF_* responsible for propulsion is a result of the interaction between the emitted acoustic field and the surrounding fluid. As given by the following equation, it can be expressed as the sum of the acoustic radiation force *F_ARF_* that presents the main force in action, the acoustic streaming force *F_AS_* and the secondary effects, such as viscous and cavitation forces, *F_other_*:

(1) $$F_{ADF}=F_{ARF}+F_{AS}+F_{other}$$

However, since the acoustic streaming force and the secondary effects, such as viscous and cavitation forces, are negligible compared to the contribution of the acoustic radiation force, the total force can be approximated as follows:

(2) $$F_{ADF}\approx F_{ARF}$$

*F_ADF_* can therefore be calculated based on the acoustic radiation pressure *P_ARF_*. Power density Π of the acoustic field is given by the sum of kinetic and internal energy components as follows:

(3) $$\Pi =\frac{1}{2}\;\rho v^{2}+\rho \varepsilon$$

where 𝜌 is the total fluid density, 𝑣 is the acoustic particle velocity, and 𝜀 is the internal energy per unit mass. Considering the fluid compressibility, internal energy 𝜌𝜀 can be expressed as^[^ ^43^ ^]^

(4) $$\rho \varepsilon =\rho_{0}\varepsilon_{0}+\left(\varepsilon_{0}+\frac{P_{0}}{\rho_{0}}\right)\rho^{\prime }+\frac{c^{2}}{2\rho_{0}}{\rho^{\prime }}^{2}$$

where *ρ′* is the density fluctuation,*P*_0_ is the ambient pressure, and *c* is the speed of sound in the fluid. As a result, the acoustic radiation pressure can be derived by time‐averaging the power density:

(5) $$P_{ARF}=\langle \frac{1}{2}\rho \;v^{2}+\frac{c^{2}}{2\rho_{0}}\;{\rho^{\prime }}^{2}\rangle$$

In the case of a Bessel acoustic vortex beam, the acoustic pressure field is given in cylindrical coordinates (*r*, ϕ, *z*) by

(6) $$p\left(r,\phi ,z\right)=P_{a}\;J_{l}\left(k_{r}r\right)e^{i\left(l\phi +k_{z}z-\omega t\right)}$$

where *P_a_* is the pressure amplitude, *J_l_* is the Bessel function of order 𝑙 representing the topological charge number of the vortex, *k_r_* and *k_z_* are the radial and axial wave numbers such that *k* = *k_r_* ^2^+*k_z_* ^2^, and ω is the angular frequency. The corresponding particle velocity field *v* can be defined as:

(7) $$v=-\frac{1}{i\omega \rho_{0}}\;\nabla p$$

Unlike a plane wave, the vortex beam creates asymmetric pressure and velocity distributions due to its azimuthal phase variation and annular intensity profile, which leads to a net directional propulsion force through both radiation pressure gradients and streaming flows. Thus, instead of a uniform pressure over the transducer face, the effective force is applied over the vortex beam cross‐section *A_vortex_* and the acoustic driving force becomes

(8) $$F_{ADF}=\int_{\;A_{vortex}}\;P_{ARF}\;\left(r\right)\;dA$$

where *dA* = *r* *dr* *d*ϕ. The calculation of the integration to obtain the acoustic drive force can be done using various methods, including the direct integration of the acoustic vortex field using finite element analysis or through analytically or experimentally obtained pressure fields. Finally, the reaction‐based propulsion is

(9) $$F_{AP}=-F_{ADF}$$

This reaction‐based force drives the swimmer forward, which depends on the distribution, intensity, and frequency characteristics of the acoustic vortex field.

### 2.2 Design and Working Principle of the Miniature Robotic Swimmer

Building on the above theoretical basis, the miniature robotic swimmer is designed to operate on the surface of water using FAVP to achieve various movement patterns without the need for movable parts in contact with the surrounding fluid environment, such as traditional propellers. It features a D‐shaped design for enhanced hydrodynamic performance, minimizing drag, improving stability, and providing room for onboard components for control and operation.

**Figure 1:**
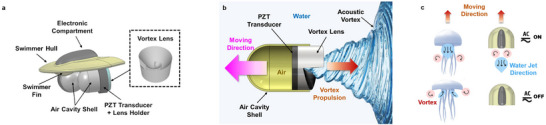
Design and working principle of the miniature robotic swimmer. a) Schematic representation of the miniature robotic swimmer. b) Principle of operation of the acoustic propulsion system using an acoustic vortex. c) Comparison of biological and the proposed vortex‐based propulsion. Left: Jellyfish generate thrust by rhythmically contracting their bell, forming vortex rings that push them forward through the water. Right: the miniature robotic swimmer utilizes an externally applied focused acoustic vortex, generating forward motion without physical appendages.

The propulsion system comprises two ultrasonic transducers, each mounted on an air‐backed cavity with a length of *L_cv_* = 10 mm and a diameter *D_cv_* = 30 mm and coupled with a 3D‐printed acoustic lens of equal diameter positioned beneath the swimmer and submerged in the water. Operating at a resonant frequency of 1 MHz, the transducers emit ultrasonic waves through the lens, forming a focused acoustic vortex that imparts OAM to the fluid. This results in localized pressure gradients and rotating flow patterns that generate a net thrust force on the swimmer's body. The interaction between the swimmer's body and the thrust force generated by the acoustic vortex further leads to translational motion, effectively propelling the swimmer across the water surface. **Figure** **1** presents a comprehensive illustration of the miniature robotic swimmer's design and its underlying acoustofluidic propulsion mechanism. The figure highlights key components of the propulsion system, including the arrangement of piezoelectric transducers, the focused acoustic vortex lens, and the thrust generation process. Additionally, it offers a comparative visualization between biological vortex propulsion as observed in jellyfish locomotion and the proposed acoustic vortex‐based propulsion. This comparison underscores how the swimmer mimics nature by utilizing vortex dynamics, offering a novel, biomimetic, and mechanically simpler approach to microscale propulsion.

The acoustic vortex lens plays a crucial role in shaping and focusing the acoustic field, thereby enhancing the stability and directionality of the propulsion. By adjusting the amplitude and phase of the emitted sound waves and the topological charge of the vortex upon design, the intensity and characteristics of the vortex can be controlled, allowing for fine‐tuning of the swimmer's speed and direction. To generate a focused acoustic vortex, the acoustic lens needs to feature a spiral phase profile, which combines an azimuthally varying thickness with concave shape properties. The variation in lens thickness introduces a phase delay as the acoustic waves propagate through it, resulting in distinct pressure fields in the far field. The thickness distribution at different locations of the lens can be determined by relating it to the phase profile of the vortex beam, ϕ(*r*, θ) as follows^[^ ^44^, ^45^, ^46^ ^]^:

(10) $$\phi \left(r,\theta \right)\;=\left[k_{m}\left(\sqrt{r^{2}+f^{2}}-f\right)+l\;\theta \right]mod\;2\pi ,\;\;\phi \left(r,\theta \right)\in \left[0,2\pi \right]$$

(11) $$h\left(r,\;\theta \right)=h_{0}+\frac{\phi \left(r,\theta \right)}{k_{m}-k_{l}}$$

where *h*(*r*,  θ) denotes the thickness of the lens at the point defined by the polar coordinates (*r*, θ), the parameter *h*_0_ is the minimum thickness of the lens, ensuring a realistic structure. The terms *k_m_* and *k_l_* represent the wavenumbers in the surrounding medium and the lens material, respectively. The vortex topological charge is donated by *l*, and *f* is the focal length of the lens. Equations (10) and (11) enable the creation of the desired focused spiral phase profile, which varies from 0 to 2π in the plane perpendicular to the propagating direction. To simplify the 3D printing process, an initial base height, *h*_0_, of 4 mm was chosen. This initial height led to a corresponding maximum height of *h_max_* = 7.47 mm.

### 2.3 Numerical Simulation

**Figure 2:**
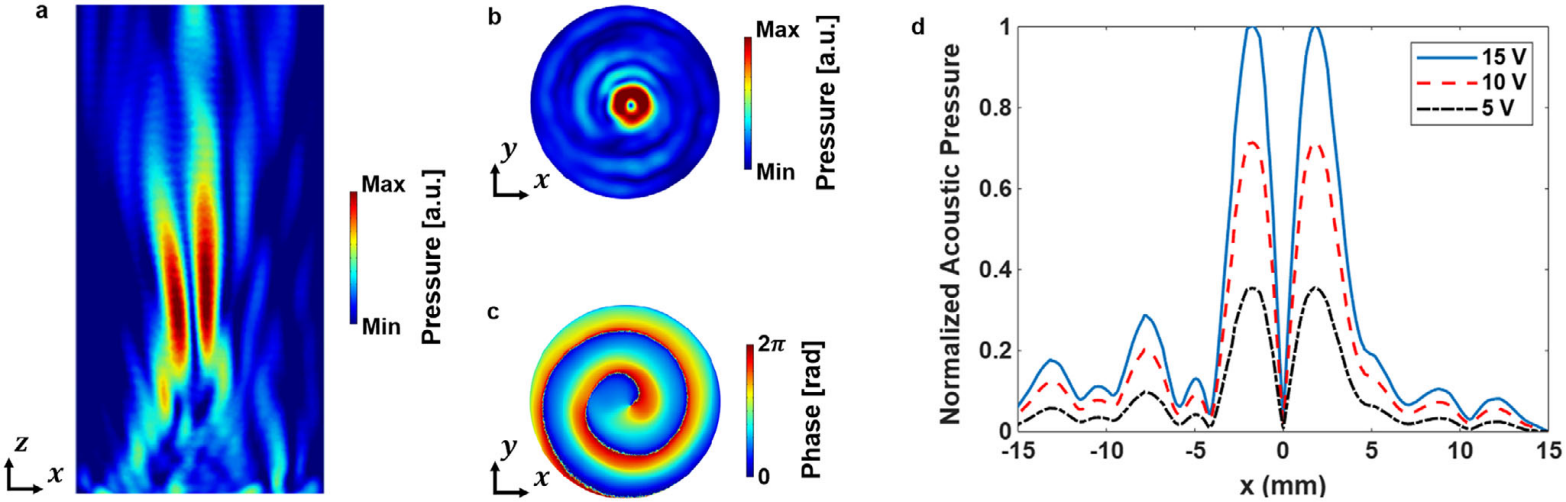
Numerical simulation results of the proposed focusing acoustic vortex lens. a) Normalized profile pressure in the xz plane. b) Normalized cross‐sectional distributions of pressure at the focal plane z = 30 mm. c) Cross‐sectional phase distribution at the focal plane z = 30 mm. d) Normalized cross‐sectional distributions of pressure at the focal plane z = 30 mm for various applied voltages.

To assess the effectiveness of the propulsion system in generating the necessary thrust for the miniature robotic swimmer, finite element simulations were conducted using COMSOL Multiphysics software. The simulation model featured a lens with a total diameter of 30 mm, which was fully enclosed within a cylindrical space measuring 60 mm in height and 30 mm in diameter, concentric with the lens. To simulate the PZT transducer attached to the vortex lens, a 30 mm diameter piezoelectric crystal bonded to a brass membrane was included to induce bending in the membrane and generate acoustic pressure. An alternating current (AC) electric potential was applied to the upper surface of the crystal, while the bottom surface was grounded. A linear elastic model for the fluid was used in the simulation. Given the small attenuation in the lens material and the background medium, loss is not considered in the numerical model.^[^ ^42^ ^]^ All the media were considered to be linear and isotropic in the simulation. The density and speed of sound assigned to the acoustic lens and surrounding medium were based on the properties of cured SLA resin and water, respectively, and are ρ_*l*_ = 1178 kg m^−3^, *c_l_* = 2591 m s^−1^ and ρ_*m*_ = 1000 kg m^−3^, *c_m_* = 1490 m s^−1^.^[^ ^47^ ^]^ For the PZT crystal, the material properties were density ρ_*pzt*_ = 7800 kg m^−3^, Young modulus *E_pzt_* = 66.6 GPa and a Poisson's ratio $\upsilon_{pzt}$ = 0.33 while for the brass membrane it has a density ρ_*b*_ = 8900 kg m^−3^, Young modulus *E_pzt_* = 100 GPa and a Poisson's ratio $\upsilon_{pzt}$ = 0.3.^[^ ^48^ ^]^ The equation that formed the basis of the numerical analysis uses the inhomogeneous Helmholtz equation. For a lossless adiabatic flow, it reads

(12) $$\Delta \cdot \left(-\frac{1}{\rho }\Delta p_{t}\right)-\frac{{k_{eq}}^{2}p_{t}}{\rho }=0$$

here, the material density is donated by *ρ*, the total pressure is presented by *p_t_*, while *k_eq_* represents the wave number that contains both the ordinary wave number *k* = $\frac{\omega }{c_{l}}$ as well as the out‐of‐plane wave number *k_z_*, where *ω* is the angular frequency and *c_l_* is the lens speed of sound. To study the wave field generated by the lens, the PZT transducer was excited at an acoustic excitation frequency of 1 MHz at the lens's base. Impedance boundary conditions were placed along the cylindrical area's perimeter and apex to eliminate unwanted reflections during the simulation. The focused acoustic vortex lens had a focal length *f* = 30 mm. As shown in **Figure** **2**, the simulation results at the focal length of the lens revealed that the variations in phase and amplitude were consistent with anticipated outcomes drawn from both theoretical insights and existing literature on focused acoustic beam vortices.^[^ ^49^, ^50^ ^]^ In addition, a higher electric voltage applied to the transducer at the bottom of the lens will lead to a more pronounced pressure field distribution as shown in Figure 2d. The ability to precisely control the intensity of the vortex pressure is important in controlling the transfer of the orbital angular momentum generated by the vortex and the associated motion, as will be demonstrated in the next section.

## 3 Experimental Results

### 3.1 The FAVP System

**Figure 3:**
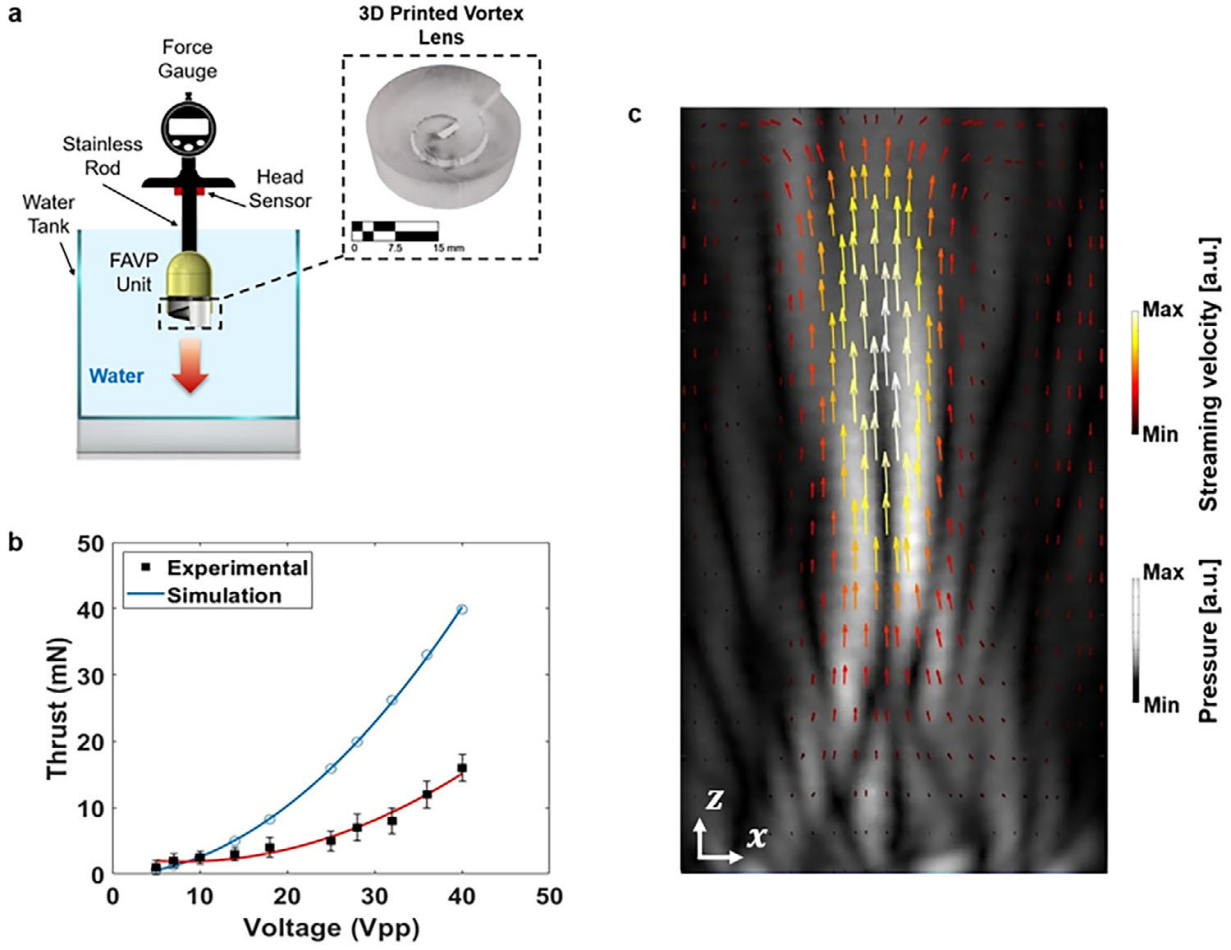
Propulsion force and the streaming velocity field induced by the focused acoustic vortex beam. a) Schematic diagram of the ZSP measurement setup with the printed acoustic vortex lens. b) The result of the acoustic vortex propulsion: simulation data (blue curve) and ZSP experimental measurement (markers) with a quadratic fit (red curve) applied to the measured data for input voltage at 1 MHz. c) Numerical simulation of the streaming velocity field along the xz plane, where the velocity increases toward the focal point and gradually decreases as the distance from the focal point increases.

To evaluate the performance of the proposed FAVP system driving the miniature robotic swimmer, a prototype lens was fabricated using stereolithography (SLA) 3D printing with a print resolution of 50 µm. This resolution ensured smooth lens surfaces and minimized printing defects. The lenses had a consistent diameter of 30 mm, matching the transducer size. The lenses featured a base height of 4 mm and a peak height of 7.47 mm, aligning with the previously defined geometry. The zero‐speed propulsion (ZSP),^[^ ^51^ ^]^ a critical performance metric for vortex propulsion systems, was measured using a ZMF‐5N force gauge, as can be seen in **Figure** **3**a. The FAVP unit was securely fixed to the force gauge, ensuring that the entire vortex propulsion system remained stationary during measurements. This configuration minimized external influences such as fluid resistance, wire traction, and other mechanical effects, providing reliable ZSP force data for performance evaluation.

Figure 3b presents the experimentally measured and numerically simulated ZSP thrust forces as a function of transducer voltage. The corresponding simulated streaming velocity distribution in the xz plane generated by the focused acoustic vortex lens is illustrated in Figure 3c. The results demonstrated that as the input voltage to the transducer increased, the generated propulsion also increased, exhibiting a quadratic relationship between the applied voltage and the propulsion force. This relationship was confirmed by both experimental measurements and numerical simulations. On the other hand, discrepancies between the experimental and simulated thrust values were observed despite the similar overall trend. These differences are primarily attributed to factors such as wave refraction caused by the lens, as well as energy losses during vortex formation and the transfer of OAM to the surrounding water, which reduced the overall performance of the lens and thus the measured force.

### 3.2 The Miniature Robotic Swimmer

To assess the swimming behavior of the miniature robotic swimmer, a prototype was fabricated using an SLA 3D printer. The swimmer's dimensions were 70 mm in length and 80 mm in width. After the 3D printing process, two units of the FAVP system, each equipped with a 1 MHz transducer (SMR, Davenport, FL), were integrated beneath the swimmer's hull. These transducers were positioned at a 90° angle relative to each other to facilitate thrust vectoring, a critical mechanism for controlling the swimmer's 2D movement. Each transducer had a diameter of 30 mm and a thickness of 2 mm. The FAVP units were fully submerged in water, while the swimmer's hull remained buoyant at the water's surface. The relatively large dimensions of the prototype miniature robotic swimmer primarily stem from the size of the selected 30 mm diameter transducer. Choosing these commercially available transducers allowed for straightforward assembly and reliable testing of the propulsion system's principles. Importantly, this scale facilitates ease of fabrication, precise control, and robust experimental validation. However, the fundamental FAVP system architecture is inherently scalable, by utilizing smaller transducers and correspondingly miniaturized components, the entire swimmer can be downsized to millimeters range. Such scaling does not compromise the propulsion concept or the control mechanisms, enabling the future development of significantly smaller swimmers tailored for applications requiring reduced dimensions.

**Figure 4:**
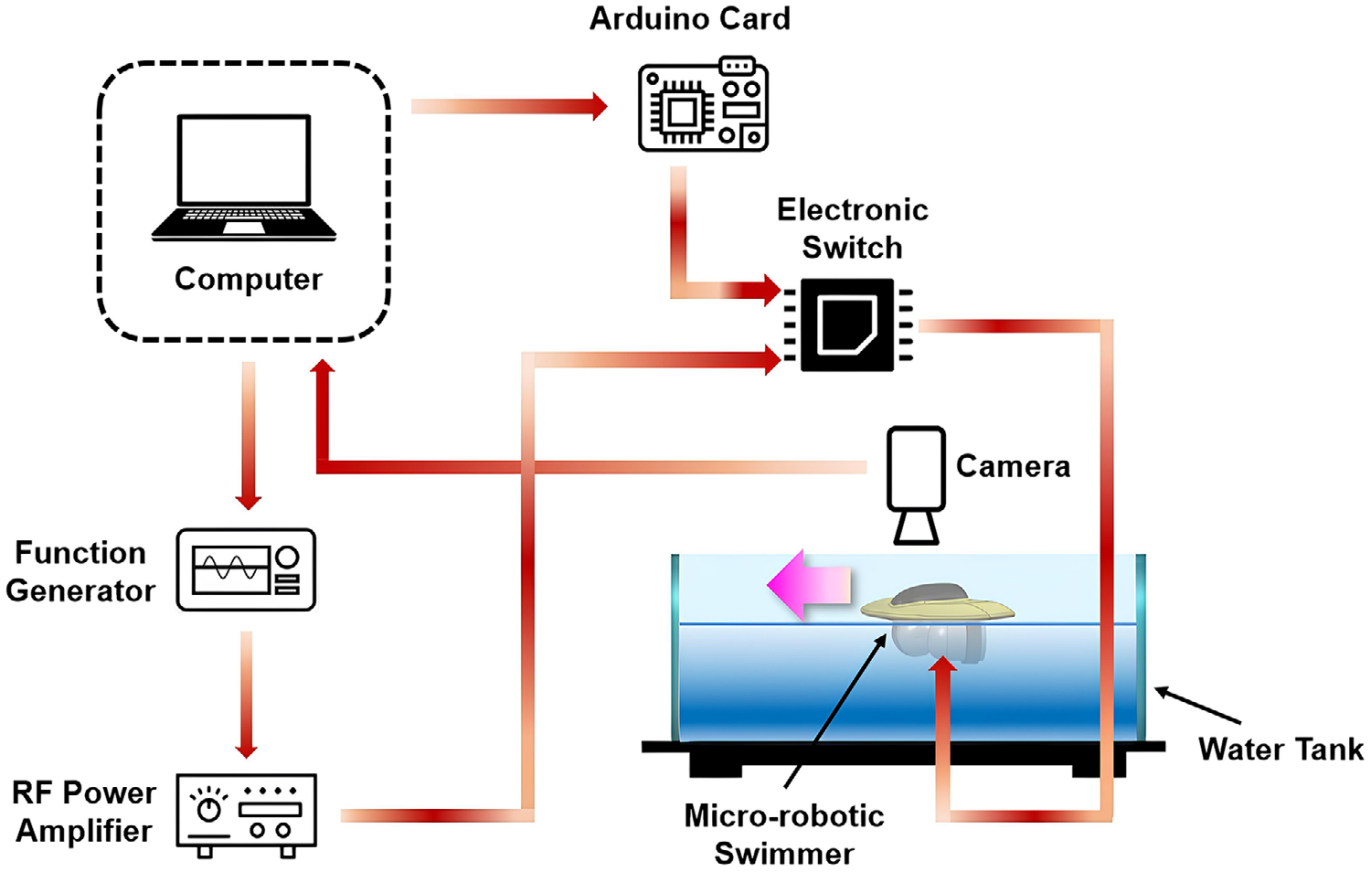
Schematic of the experimental setup used for the miniature robotic swimmer testing.

To regulate the thrust generated by each propulsion unit and ensure synchronized operation, both FAVP units were connected to a microcontroller (Arduino Nano, Belmont, CA) via dedicated input pins. A custom‐written program was developed to control the operation of the FAVP system, enabling precise manipulation of the swimmer's trajectory in the xy plane and providing controlled movement of the robotic swimmer. A function generator (RIGOL DG4162, Portland, OR) was used to produce a sinusoidal signal, which was subsequently amplified by an RF power amplifier (ENI 3200L, Renton, WA) to achieve the requisite acoustic wave intensity at the base of the vortex lenses. Given that the voltage range required to drive the FAVP system exceeds the maximum output capacity of the microcontroller output pins, a non‐mechanical electronic switch was incorporated into the system. This component served to control the flow of the RF power amplifier's output to the FAVP units, in coordination with the microcontroller commands, thus ensuring effective power management and control over the swimmer's propulsion (**Figure** **4**).

**Figure 5:**
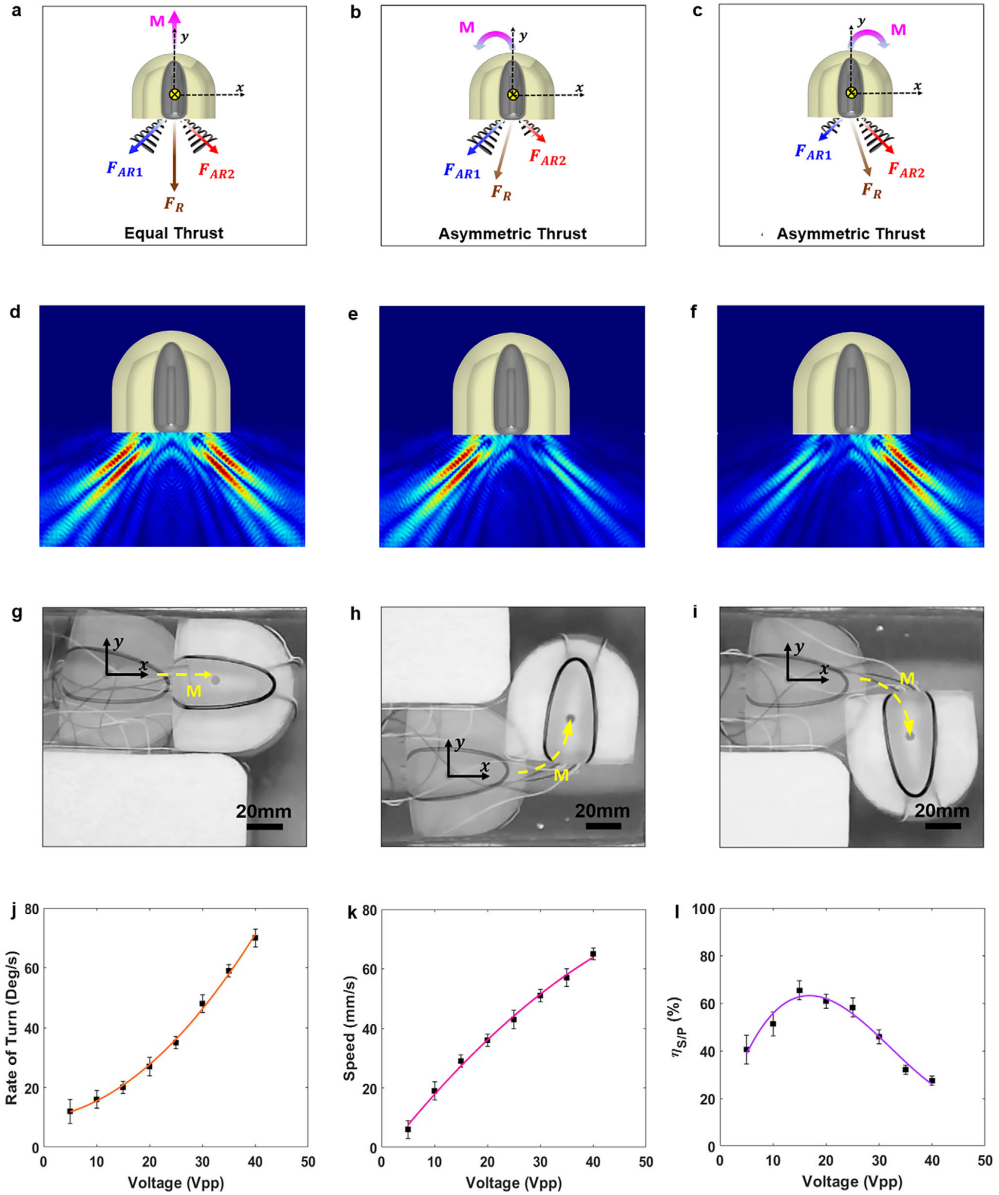
Generated movement based on the status of the FAVP units and their performance characterization. a–c) Schematic of different translational and rotational movements of the miniature robotic swimmer based on FAVP status. d–f) Numerical simulation of the different translational and rotational movements based on FAVP status. g–i) Experimental validation of the different translational and rotational movements based on FAVP status. j–l) Rate of turn, speed, and speed to power ratio efficiency η_*S*/*P*_ of the proposed miniature robotic swimmer as a function of voltage.

To evaluate the motion performance of the proposed miniature robotic swimmer, a high‐resolution wide‐field video camera (Logitech, San Jose, CA) was mounted above the water tank, capturing frames at 10 Hz. A magenta color tracking marker was affixed to the swimmer hull for motion tracking and analysis. In the absence of actuation (0 Vpp), the swimmer remained stationary at the water's surface. Upon execution of the control sequence via the microcontroller, the swimmer achieved a peak velocity of 67 mm s^−1^, driven by full activation of the FAVP system. This system enabled navigation along preprogrammed trajectories at the water surface. The swimmer's motion was governed by a discrete sequence of voltage inputs to each of the two independent FAVP units, generating variable thrust conditions: full thrust (40 Vpp), zero thrust (0 Vpp), or modulated thrust depending on the specified pattern or maneuver. By independently controlling each transducer, the system achieved thrust vectoring, enabling both translational and rotational movements with precision. The principle of motion generation, along with numerical simulations and corresponding experimental results, is presented in **Figure** **5**a–i respectively.

Additionally, Figure 5j,k further characterize the swimmer's turning rate and instantaneous velocity as functions of the applied voltage. These results demonstrate that increased input voltage leads to proportionally greater angular and linear velocities, reflecting the FAVP system's tunable dynamic response. To assess propulsion efficiency, the speed‐to‐power ratio η_*SP*_ defined as the swimmer's velocity normalized by the input electrical power, was measured and is shown in Figure 5l. As expected, η_*S*/*P*_ decreases with increasing voltage due to the nonlinear behavior of power consumption, indicating diminishing returns in propulsion efficiency at higher inputs. Conversely, at lower voltages, higher η_*S*/*P*_ values underscore efficient energy to motion conversion, a desirable trait for energy‐constrained applications. This voltage‐dependent trade‐off between speed and energy efficiency is crucial for optimizing endurance and maneuverability in real‐world scenarios. Identifying an operational regime with maximal η_*S*/*P*_ provides a pathway to sustained, controlled locomotion with minimal energy overhead for a fully autonomous system. To offer a more comprehensive characterization of the swimmer's motion capabilities, additional performance metrics are provided in Figures S1–S3 (Supporting Information). These include the minimum turning radius, which quantifies the swimmer's maneuverability limits, the cost of travel (CoT), which captures the energetic expenditure per unit distance, and the speed‐to‐thrust ratio η_*S*/*T*_, which serves as a direct indicator of propulsive efficiency across the swimmer's operating speed range. Together, these curves provide deeper insight into the swimmer's maneuverability, energy usage, and thrust effectiveness under different operating conditions.

**Figure 6:**
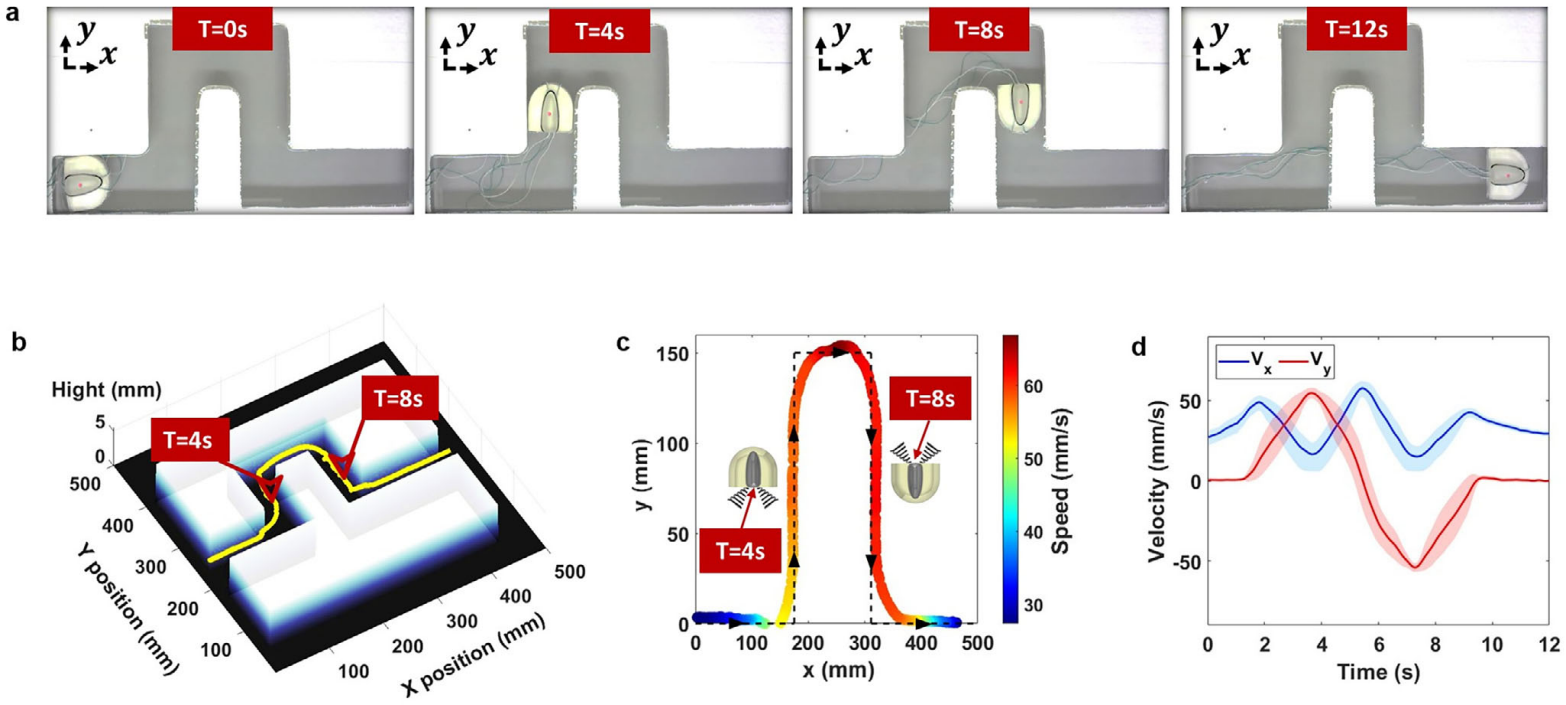
ZigzagPu motion sequence and corresponding measurement results of the miniature robotic swimmer. a) Snapshot of the miniature robotic swimmer at various time instances during its motion. b) Experimental tracked trajectory of the miniature robotic swimmer superimposed on the corresponding obstacle map. c) Comparison between the experimental trajectory and the ideal programmed path, including the miniature robotic swimmer's instantaneous speed. d) Axial and tangential speed components of the miniature robotic swimmer along the trajectory, with shaded envelopes indicating the standard deviation across multiple trials.

The swimmer's performance was further validated through experimental trials in a water tank environment, where two complex trajectories, namely a zigzag path and a letter “R” shape, were executed. Full video recordings of both trajectories are available in Videos S1 and S2. For the zigzag trajectory, time‐lapse images at t = 0, 4, 8, and 12s are shown in **Figure** **6**a. To assess repeatability, each trajectory was executed three times. A custom MATLAB tracking algorithm, utilizing color thresholding and geometric detection, was used to extract the swimmer's path, superimposed on the corresponding obstacle map (Figure 6b). Figure 6c presents the experimentally measured trajectory and instantaneous velocity for a representative trial, overlaid with the programmed path (dashed line). Velocity varied dynamically based on maneuver complexity, ranging from 30 mm s^−1^ to a maximum of 67 mm s^−1^. Axial *V_x_* and tangential *V_y_* velocity components for the same trial are plotted in Figure 6d, with shaded regions indicating standard deviations across all trials, demonstrating the swimmer's consistent performance. Quantitatively, the miniature swimmer exhibited a positional accuracy within a boundary tolerance of ∓ half the width of the swimmer relative to the centerline of 90.83% and a speed precision with respect to the programmed speed sequence of 83.39% during the zigzag trials, highlighting the effectiveness of the proposed approach.

**Figure 7:**
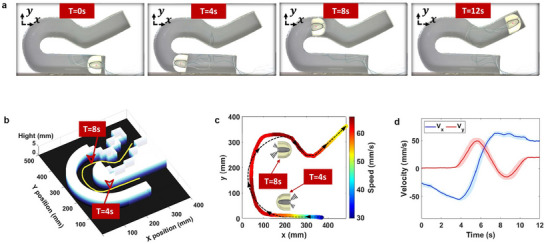
Letter “R” shape motion sequence and corresponding measurement results of the miniature robotic swimmer. a) Snapshot of the miniature robotic swimmer at various time instances during its motion. b) Experimental tracked trajectory of the miniature robotic swimmer superimposed on the corresponding obstacle map. c) Comparison between the experimental trajectory and the ideal programmed path, including the miniature robotic swimmer's instantaneous speed. d) Axial and tangential speed components of the miniature robotic swimmer along the trajectory, with shaded envelopes indicating the standard deviation across multiple trials.

In the same fashion, the experimental measurements of the miniature robotic swimmer movement following a letter “R” shaped pattern are shown in **Figure** **7**a through snapshots taken at t = 0 s, t = 4s, t = 8 s, and t = 12 s, respectively. Similar to the previous set of trials, three repetitions were conducted to ensure reliable performance. The tracked locomotion of the swimmer during a selected trial with respect to the geometry of the obstacle is shown in Figure 7b. The experimental trajectory and instantaneous speed, compared with the programmed path presented with a dashed line, are presented in Figure 7c. Figure 7d displays the swimmer's axial *V_x_* and tangential *V_y_* speed components for the selected trial. The results again confirm the repeatability and consistency in regulating complex movements in water. Furthermore, for the Letter “R” shape motion sequence, the swimmer achieved a positional accuracy within the boundary tolerance relative to the centerline of 94.74% and a speed precision with respect to the programmed speed sequence of 88.61%, further confirming its reliable and precise actuation capability.

**Figure 8:**
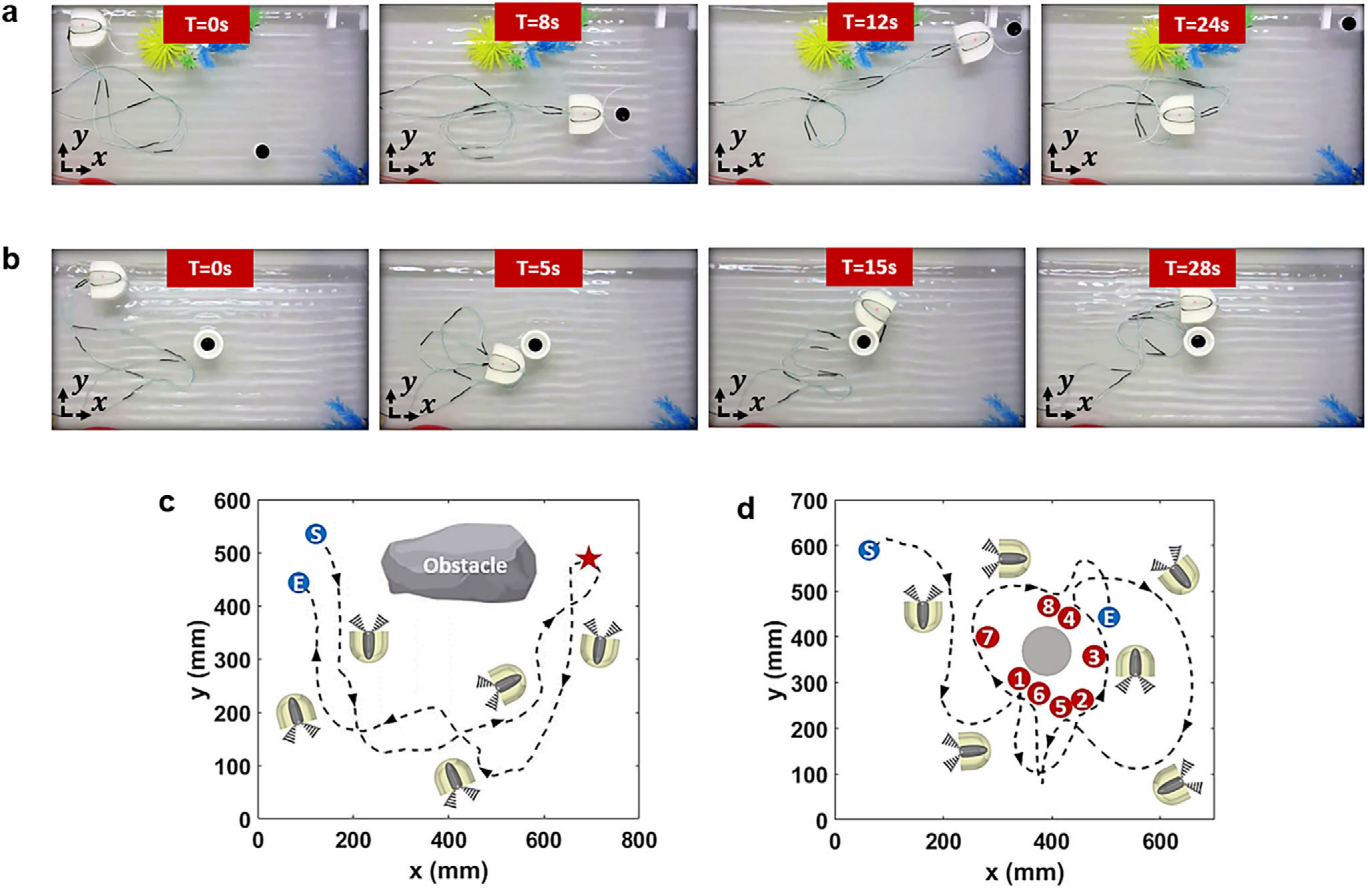
Task‐oriented experiments in realistically disturbed aquatic environments. a) Snapshots of the robotic swimmer during the object‐transport mission. b) Snapshots during the structural‐inspection mission. c) Tracked trajectory of the object‐transport mission: blue “S” and “E” indicate start and end points, red star marks the object delivery location. d) Tracked trajectory of the structural‐inspection mission: blue “S” and “E” indicate start and end points, red numbered circles mark stop points for simulated data collection.

To further evaluate the swimmer's performance under realistic environmental disturbances and mission‐based conditions, two additional task‐oriented experiments were conducted (see Videos S3 and S4). The first task‐oriented experiment demonstrates an object‐transport mission performed in open water while the tank is subjected to perturbations from mechanical waves. These waves were generated using an electromechanical vibratory actuator driven by a programmable waveform generator, producing a continuous 10 Hz oscillatory motion at one side of the tank to emulate non‐stagnant flow and mimic a realistic environment. Under these conditions, the swimmer successfully grasps a passive object, navigates around an obstacle, delivers it to a designated target location at the other side of the water tank, and subsequently returns to its initial position. **Figure** **8**a presents snapshots of the preformed experiment taken at t = 0 s, t = 8 s, t = 12 s, and t = 24 s, respectively. Figure 8c depicts the tracked trajectory of the swimmer during the experiment. The recorded trajectory confirms that the swimmer maintains a stable overall trajectory despite the persistent wave‐induced perturbations, indicating the effectiveness of the acoustic vortex propulsion method. Following the same approach, the second experiment involves mimicking a structural‐inspection mission in which the swimmer circumnavigates a central beam and stops at eight inspection spots distributed around the structure. These locations collectively cover all surrounding directions, simulating a multi‐angle data‐collection or sensing task where the swimmer would pause to capture information at each position. The swimmer completes the task by exhibiting controlled yaw rotation and stable translation as it approaches and leaves each inspection point. Figure 8b presents snapshots of this mission at t = 0 s, 5 s, 15 s, and 28 s, and the corresponding tracked trajectory is shown in Figure 8d. These results highlight the swimmer's ability to perform complex, mission‐critical tasks with precision and stability in realistically disturbed aquatic environments.

Some deviations between the experimentally observed and preprogrammed paths were also observed during the measurements, especially in the case of the task‐oriented open water experiments. Specifically, the executed trajectories did not consistently replicate the programmed sequences, and variations in instantaneous velocity were recorded throughout the trials. These discrepancies are attributed to a combination of experimental constraints and system‐level factors. Despite the use of flexible electric wires, residual mechanical tension exerted by the tether introduced perturbations in the swimmer's motion, contributing to trajectory deviations and speed fluctuations. To mitigate this effect, an obstacle‐guided path was implemented in the first two sets of experiments (zigzag and “R” shaped trajectory), acting as a physical constraint to stabilize motion and minimize tether‐induced artifacts while preserving the swimmer's maneuverability. Additional sources of deviation included fluctuations in power delivery to the FAVP system, which affected actuator response, and increased hydrodynamic drag arising from surface roughness on the swimmer's hull, particularly during high‐curvature maneuvers. These factors contributed to discrepancies between the predicted and observed trajectories. To address these limitations, future iterations of the system could benefit from incorporating closed‐loop feedback control, such as proportional‐integral‐derivative (PID) algorithms, to dynamically correct for real‐time deviations in trajectory and velocity, especially for a disturbed open water environment.^[^ ^52^, ^53^ ^]^ Moreover, fully embedding the control system on the hull itself and optimizing the fabrication process to produce smoother, hydrodynamically efficient hull surfaces will help reduce drag and enhance stability while completely eliminating any wire tensions. These improvements are expected to significantly increase trajectory fidelity, energy efficiency, and overall robustness by creating a full autonomous system, thereby advancing the swimmer's applicability in precision‐demanding environments.

## 4 Discussion and Future Directions

The FAVP method demonstrated in this study enables precise 2D locomotion of a surface‐bound robotic swimmer, unlocking exciting possibilities for controlled small‐scale movement within fluid environments. This capability positions the swimmer as a valuable tool in a variety of settings. For instance, in laboratory and industrial fluid‐handling systems, it could be used to accurately position sensors, collect samples, or interact with floating elements within reaction chambers or tanks. Likewise, in controlled aquatic environments such as aquaculture facilities, biomedical research platforms, or chemical analysis setups, the swimmer's ability to perform repeated, patterned tasks across defined surface areas can reduce the need for manual intervention.Moreover, the swimmer unique mechanical simplicity, which achieves forward motion, turning, and directional control without any moving parts contacting the fluid, makes it especially suited for sensitive or sterile environments where minimizing turbulence and contamination is paramount. Additionally, the dual‐thruster design opens the door to multi‐agent coordination, enabling spatial patterning and distributed surface scanning on shared water surfaces in contained systems.

Looking ahead, a key avenue for future development lies in achieving wireless actuation and control. Currently, the prototype relies on wired connections to supply the needed power to the ultrasonic transducers, limiting operational flexibility. However, rapid advances in miniaturized electronics and power technologies suggest promising pathways toward fully integrated, untethered systems. Embedding compact control units with onboard power supplies would enable autonomous propulsion and maneuvering without physical constraints, which are a critical requirement for deployment in complex or confined environments, such as biomedical applications, environmental monitoring, or coordinated robotic swarms. Wireless acoustic vortex propulsion thus represents a natural and compelling extension of this work, poised to unlock new functionalities and broaden the range of practical applications.

Furthermore, although our prototype was developed at the centimeter scale to leverage commercially available transducers for experimental validation, the underlying propulsion architecture is inherently scalable down to the millimeter range. Miniaturization, however, introduces a series of technical challenges that must be addressed to maintain effective performance. As acoustic lenses and transducers shrink, the acoustic energy transmitted into the fluid diminishes, potentially reducing thrust. To compensate, strategies such as utilizing higher‐frequency transducers, optimizing lens geometries, and employing materials with superior acoustic transmission properties will be essential. Additionally, at smaller scales, surface forces including drag and surface tension become increasingly significant, necessitating careful geometric optimization to ensure hydrodynamic stability and minimize resistance. Precision in fabrication also grows more critical, as even slight misalignments can substantially degrade acoustic focusing and thrust generation. Encouragingly, recent advances in microfabrication techniques, MEMS ultrasonic transducers, and lightweight materials offer promising solutions to these challenges, paving the way toward compact, efficient acoustic vortex swimmers.

Another important aspect of this propulsion method is its operation at ultrasonic frequencies well beyond the hearing range of humans and most animals, rendering it effectively silent in marine environments. The localized concentration of acoustic energy at the vortex focal point further minimizes acoustic leakage and unwanted disturbance. Nonetheless, in highly sensitive contexts such as biomedical applications or ecologically fragile habitats, even small acoustic emissions could raise concerns. Future designs can address this by incorporating mitigation strategies, including reducing power output, limiting duty cycles, or employing acoustic shielding, thereby adapting the system for use in delicate environments without compromising performance.

While the current prototype operates under open‐loop control to demonstrate fundamental actuation and maneuvering, the dual‐thruster configuration establishes a strong foundation for implementing closed‐loop control. Integrating real‐time tracking via optical sensors or sensor fusion would enable continuous monitoring of the swimmer's position and orientation, allowing dynamic adjustment of actuation signals. This closed‐loop feedback would support autonomous trajectory correction, target following, and enhanced motion precision, which are particularly valuable in complex or dynamic fluidic environments. Future work will focus on developing these feedback control systems to unlock advanced functionalities such as swarm coordination and autonomous task execution in biomedical and environmental contexts.

Finally, while our experiments have thus far been limited to controlled aquatic environments, extending the system to operate in open outdoor or semi‐structured water bodies presents an important next step. These real‐world settings introduce additional challenges, including currents, waves, and environmental noise, all of which demand robust trajectory tracking and adaptive control strategies. Advanced localization and feedback mechanisms will be critical to ensuring precise navigation and maneuvering under such conditions, thereby significantly broadening the system's practical applicability to fields such as environmental monitoring, aquatic exploration, and beyond.

## 5 Conclusion

This study introduces a novel and effective approach to the propulsion and control of miniature robotic swimmers using a proposed FAVP system based on acoustic waves. By leveraging the unique characteristics of acoustic vortex lenses, the proposed method enables stable and precise movement of miniature robotic swimmers. The use of two perpendicularly positioned FAVP systems, along with differentiating their output power, allows the generation of both translational and rotational movements. Consequently, precise 2D locomotion in small‐scale swimmers is achieved, allowing complex maneuvers. Experimental validation, supported by numerical simulations, demonstrated the FAVP system's ability to generate adequate thrust to propel the swimmer at speeds of up to 67 mm s^−1^. Complex motions can also be realized by sequencing the translational and rotational movements. Additionally, the system offers the flexibility to adjust the swimmer's velocity based on specific maneuvering requirements, underscoring its versatility.

A key advantage of this approach lies in its inherent scalability. The compact and contactless nature of the FAVP system enables the design and operation of swimmers at significantly reduced sizes, down to the microscale, with appropriate size transducers. This scalability opens up possibilities for deploying such swimmers in confined or delicate environments, such as small fluidic systems, where traditional propulsion mechanisms may not be feasible. The results highlight the substantial potential of the FAVP concept for non‐contact propulsion in mini‐robotic applications. This approach offers various advantages in terms of precision, efficiency, maneuverability, and payload, presenting a strong case for its future use in a variety of applications, such as environmental monitoring and target payload delivery. Looking ahead, the study suggests several avenues for further improvement. Key developments include enhancing the control system, e.g., by integrating PID controllers, and refining the swimmer's hull design to minimize hydrodynamic drag and optimize performance. These advancements, combined with continued refinement of the FAVP system, will pave the way for the creation of highly precise, versatile, and efficient mini‐robots. In conclusion, this research represents a significant step forward in the development of non‐contact, high‐precision robotic movement. It provides a solid foundation for future innovations in the design, control, and application of mini‐robots powered by acoustic wave propulsion, with far‐reaching potential in diverse fields of scientific and industrial interest.

## Conflict of Interest

The authors declare no conflict of interest.

## Supporting information



Supporting Information

Supporting Information

Supporting Information

Supporting Information

Supporting Information

## Data Availability

The data that support the findings of this study are available from the corresponding author upon reasonable request.
