# Supplementary material for: Miniature Robotic Swimmer with Precise 2D Motion Control via Acoustic Vortex‐Induced Propulsion
Source: Adv Sci (Weinh). 2025 Dec 12;13(12):e15389. doi: 10.1002/advs.202515389 (PMC12948230; doi:10.1002/advs.202515389)
Supplement: Supplementary file 1 — Supporting Information [file ADVS-13-e15389-s003.docx]

**Supporting Information**

Miniature Robotic Swimmer with Precise 2D Motion Control via Acoustic Vortex-Induced Propulsion

Chadi Ellouzi, ^1^ Nicholas Andrianto, ^1^ Glen Vosgerichian, ^1^ Farhood Aghdasi, ^1^ Joshua Lloyd, ^1^ Ali Zabihi, ^1^ Chen Shen^1^*

*^1^Department of Mechanical Engineering, Rowan University, Glassboro, New Jersey 08028, USA*

***Corresponding Authors.**

[shenc@rowan.edu](mailto:shenc@rowan.edu)


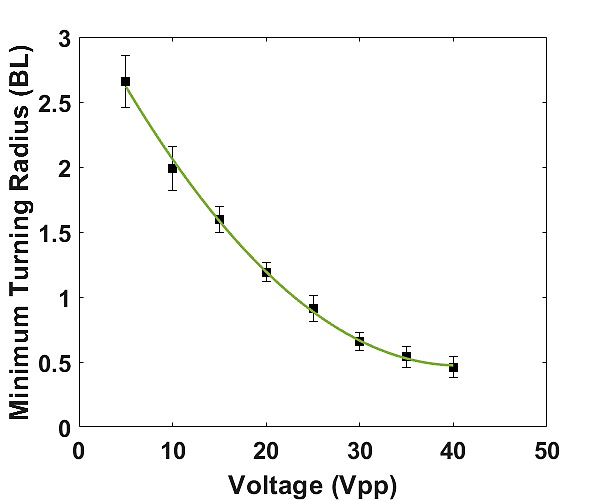


FIG.S1. Miniature robotic swimmer minimum turning radius as a function of transducer driving voltage


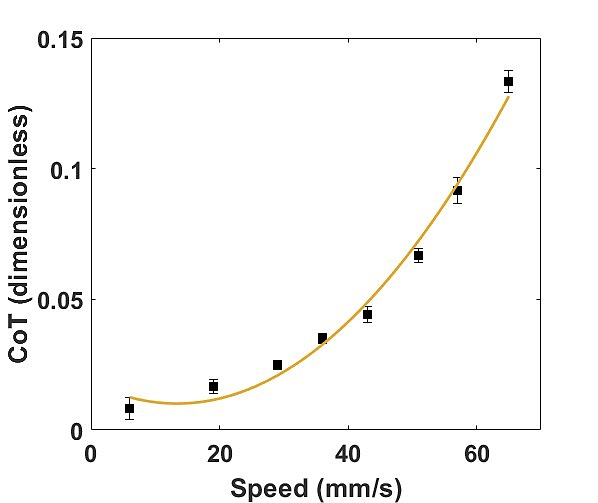


FIG.S2. Miniature robotic swimmer cost of transport as a function of the swimmer speed.


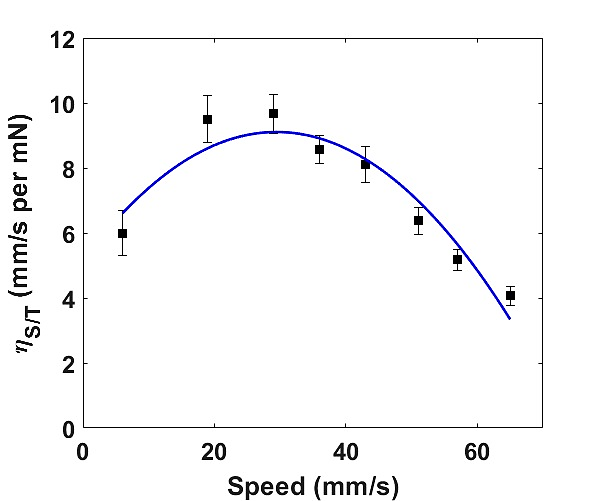


FIG.S3. Miniature robotic swimmer Speed-to-thrust efficiency ratio as a function of the swimmer speed

Table S1. Performance comparison of typical microrobots based on their propulsion type

| Propulsion Type | Max Speed ($\boldsymbol{V}_{\boldsymbol{max}}\boldsymbol{)}$  (mm/s) | Body Length ($\boldsymbol{L)}$  (mm) | Body Length Per Second  (BL/s) | Reference |
| --- | --- | --- | --- | --- |
| Acoustic | 67 | 70 | 0.96 | This Work |
| Acoustic | 27 | 60 | 0.45 | [1] |
| Acoustic | 61 | 60 | 1.01 | [1] |
| Acoustic | 66 | 47 | 1.40 | [2] |
| Acoustic | 100 | 40 | 2.50 | [3] |
| Elastic | 15 | 30 | 0.50 | [4] |
| Elastic | 85 | 30 | 2.83 | [4] |
| Shape Memory | 112 | 126 | 0.88 | [5] |
| Magnetic | 47 | 58 | 0.81 | [6] |
| Magnetic | 0.6 | 0.5 | 1.2 | [7] |
| Magnetic | 0.8 | 1 | 0.8 | [8] |
| Magnetic | 1.5 | 2.5 | 0.6 | [9] |
| Magnetic | 0.8 | 1.5 | 0.53 | [10] |
| Electrical | 27 | 80 | 0.34 | [11] |
| Light | 2.8E-3 | 1.23 | 2.28E-3 | [12] |
| Optothermal | 10E-3 | 2E-3 | 5 | [13] |

**References:**

1. Wang, Q.; Li, Z.; Bowen, C.; Courtney, C.; Pan, M.; Xu, Q.; Chen, W.; Fieldhouse, S.; Wan, C. Enhanced Buoyancy and Propulsion in 3D Printed Swimming Micro-Robots Based on a Hydrophobic Nano-Fibrillated Cellulose Aerogel and Porous Lead-Free Piezoelectric Ceramics. *Nano Energy* 2024, *131*, 110254, doi:10.1016/j.nanoen.2024.110254.

2. Luo, T.; Wu, M. Biologically Inspired Micro-Robotic Swimmers Remotely Controlled by Ultrasound Waves. *Lab Chip* 2021, *21*, 4095–4103, doi:10.1039/D1LC00575H.

3. Kong, D.; Nishio, K.; Kurosawa, M.K. Surface Acoustic Wave Propulsion System with Acoustic Radiation Force. *Sens Actuators A Phys* 2020, *309*, 111943, doi:10.1016/j.sna.2020.111943.

4. Toennies, J.L.; Tortora, G.; Simi, M.; Valdastri, P.; Webster, R.J. Swallowable Medical Devices for Diagnosis and Surgery: The State of the Art. *Proc Inst Mech Eng C J Mech Eng Sci* 2010, *224*, 1397–1414, doi:10.1243/09544062JMES1879.

5. Wang, Z.; Wang, Y.; Li, J.; Hang, G. A Micro Biomimetic Manta Ray Robot Fish Actuated by SMA. In Proceedings of the 2009 IEEE International Conference on Robotics and Biomimetics (ROBIO); IEEE, December 2009; pp. 1809–1813.

6. Liu, W.; Jia, X.; Wang, F.; Jia, Z. An In-Pipe Wireless Swimming Microrobot Driven by Giant Magnetostrictive Thin Film. *Sens Actuators A Phys* 2010, *160*, 101–108, doi:10.1016/j.sna.2010.04.014.

7. Pramanik, R.; Park, M.; Ren, Z.; Sitti, M.; Verstappen, R.W.C.P.; Onck, P.R. Computational and Experimental Design of Fast and Versatile Magnetic Soft Robotic Low Re Swimmers. *Extreme Mech Lett* 2025, *78*, 102358, doi:10.1016/j.eml.2025.102358.

8. Zhang, Z.Y.; Song, Y.B.; Wang, Y.F.; Wang, C.G. Smart Helical Swimmer: Nested and Uncoiled Designs. *Int J Mech Sci* 2023, *242*, 107996, doi:10.1016/j.ijmecsci.2022.107996.

9. Pramanik, R.; Verstappen, R.W.C.P.; Onck, P.R. Nature-Inspired Miniaturized Magnetic Soft Robotic Swimmers. *Appl Phys Rev* 2024, *11*, doi:10.1063/5.0189185.

10. Giltinan, J.; Katsamba, P.; Wang, W.; Lauga, E.; Sitti, M. Selectively Controlled Magnetic Microrobots with Opposing Helices. *Appl Phys Lett* 2020, *116*, doi:10.1063/1.5143007.

11. Chen, Z. A Review on Robotic Fish Enabled by Ionic Polymer–Metal Composite Artificial Muscles. *Robotics Biomim* 2017, *4*, 24, doi:10.1186/s40638-017-0081-3.

12. Wang, W.; Duan, W.; Ahmed, S.; Mallouk, T.E.; Sen, A. Small Power: Autonomous Nano- and Micromotors Propelled by Self-Generated Gradients. *Nano Today* 2013, *8*, 531–554, doi:10.1016/j.nantod.2013.08.009.

13. Peng, X.; Chen, Z.; Kollipara, P.S.; Liu, Y.; Fang, J.; Lin, L.; Zheng, Y. Opto-Thermoelectric Microswimmers. *Light Sci Appl* 2020, *9*, 141, doi:10.1038/s41377-020-00378-5.
